# Noncanonical Reactions of Flavoenzymes

**DOI:** 10.3390/ijms131114219

**Published:** 2012-11-05

**Authors:** Pablo Sobrado

**Affiliations:** 1 Department of Biochemistry, Virginia Tech, Blacksburg, VA 24061, USA; E-Mail: psobrado@vt.edu; Tel.: +1-540-231-9485; Fax: +1-540-231-9070; 2 Virginia Tech Center for Drug Discovery, Virginia Tech, Blacksburg, VA 24061, USA

**Keywords:** flavoenyzmes, chorismate synthase, type II isopentenyl diphosphate/dimethylallyl diphosphate isomerase, UDP-galactopyranose mutase, alkyl-dihydroxyacetonephosphate synthase, non-redox reaction, novel function

## Abstract

Enzymes containing flavin cofactors are predominantly involved in redox reactions in numerous cellular processes where the protein environment modulates the chemical reactivity of the flavin to either transfer one or two electrons. Some flavoenzymes catalyze reactions with no net redox change. In these reactions, the protein environment modulates the reactivity of the flavin to perform novel chemistries. Recent mechanistic and structural data supporting novel flavin functionalities in reactions catalyzed by chorismate synthase, type II isopentenyl diphosphate isomerase, UDP-galactopyranose mutase, and alkyl-dihydroxyacetonephosphate synthase are presented in this review. In these enzymes, the flavin plays either a direct role in acid/base reactions or as a nucleophile or electrophile. In addition, the flavin cofactor is proposed to function as a “molecular scaffold” in the formation of UDP-galactofuranose and alkyl-dihydroxyacetonephosphate by forming a covalent adduct with reaction intermediates.

## 1. Introduction

Enzymes are responsible for catalyzing most of the reactions occurring in the cell and, thus, are responsible for sustaining life. Reactions required during cellular metabolism are numerous and have diverse chemistries. Substrate specificity and chemical diversity of an enzyme originates from its amino acid composition and three dimensional structure [1–3]. However, to increase the number of chemical reactions, enzymes have recruited cofactors, including metal ions such as zinc and iron, heme, pyridoxal 5′-phosphate (PLP), thiamine pyrophosphate (TPP), biotin, folate, and flavins, among others [4]. These cofactors have unique chemical properties that are exploited by enzymes during catalysis. For example, heme-containing enzymes utilize the metal iron to either bind and transport molecular oxygen, as in hemoglobin, or to form a high-valence oxo-iron intermediate capable of hydroxylating stable hydrocarbons, such as in the case of cytochrome P450 [5,6]. Although many of these cofactors are used for various reactions and are essential for cell viability, perhaps the most diverse of all cofactors are the flavins [7–10].

**Scheme 1:**
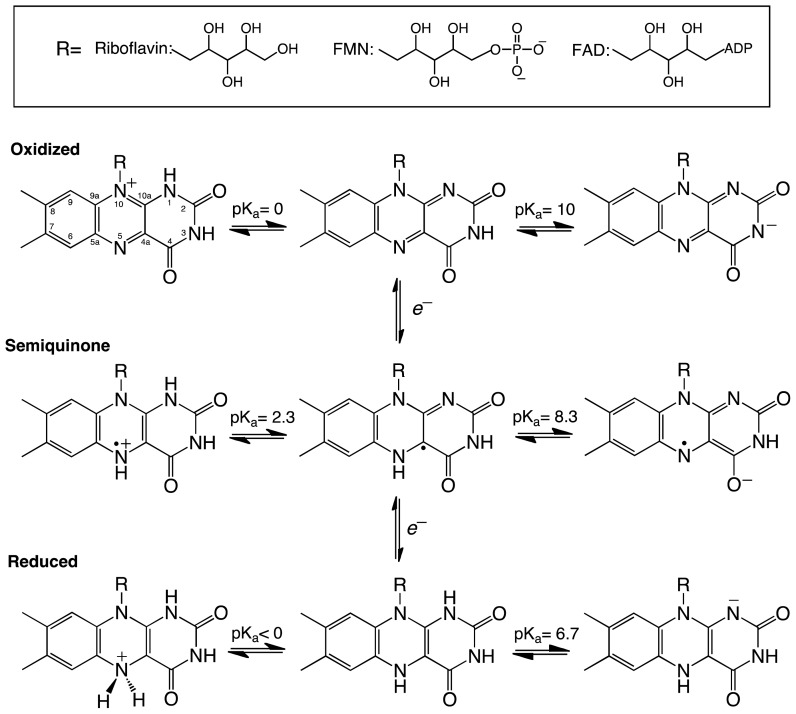
Structure and redox intermediates of flavin cofactors. Figure adapted from [11].

Flavins are derived from riboflavin (vitamin B2), which serves as the precursor for the two flavin cofactors found in eukaryotes and bacteria-flavin mononucleotide (FMN) and flavin adenine dinucleotide (FAD) [10]. The dimethyl-isoalloxazine ring of the flavin gives flavoenzymes their characteristic yellow color and, possibly, the most diverse chemical reactivity found in nature. In this three ring system, the key functional players are the N5, C4a, C10a, and N1 atoms which from the conjugated π electron system of the isoalloxazine ring. Flavins can exist in oxidized, reduced, or semiquinone forms (Scheme 1). The chemical properties of the flavin cofactor enable flavoenzymes to participate in oxidation/reduction reactions [12]. In reduction reactions, reducing equivalents are transferred to the oxidized flavin either in two electron transfer steps or in a single step as a hydride, resulting in oxidation of a substrate or coenzyme (e.g., NADPH) and reduction of the flavin. In oxidation reactions, the reduced form of the flavin transfers electrons one by one or in the form of a hydride to form the oxidized flavin and a reduced product [7,8]. For instance, in the flavin-dependent amino acid oxidases, the flavin is reduced by a hydride equivalent from the amino acid substrate and is later oxidized by molecular oxygen producing hydrogen peroxide and an imino acid intermediate that is nonenzymatically converted to the corresponding keto acid [13,14]. Flavin monooygenses (FMO) are oxidized by molecular oxygen to form a C4a-O-OH flavin intermediate, which is required for hydroxylation of the substrate [8]. FMOs represent an elegant example of the tunability of the chemical outcome of flavin-dependent reactions. These enzymes are capable of stabilizing the flavin-C4a-O-OH intermediate instead of forming hydrogen peroxide, which occurs with the flavin oxidases. The flavin-C4a-O-OH is stabilized for the hydroxylation of aromatic or aliphatic hydrocarbons, sulfur, or nitrogen containing substrates [8,15]. Deprotonation of the flavin-C4a-O-OH yields a flavin-C4a-O-O^−^ (C4a-peroxo species) capable of performing nucleophilic attack on ketones to form esters, as in the case of the Baeyer-Villiger reaction [8,12].

Apart from redox reactions, flavoenzymes are also known to catalyze reactions where no net redox change occurs; however, the flavin cofactor is required for catalysis [16]. Recent mechanistic and structural data strongly supports novel functionality of flavins in this family of enzymes, serving as either an acid or a base and in the formation of a covalent intermediate in various non-redox reactions [9]. Specifically, these are reactions where a flavin is proposed to play a mixed role, such as both an acid and a base, in electron transfer and acid/base reactions, and as a molecular scaffold forming a covalent intermediate (Table 1). Herein, a review of the structural and mechanistic data in support of these noncanonical flavin reactions is presented.

## 2. Chorismate Synthase

**Figure 1:**
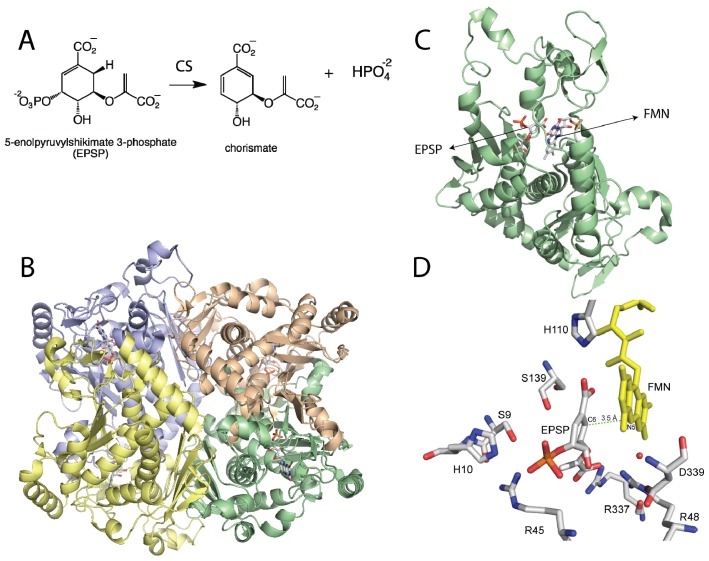
(**A**) Reaction catalyzed by chorismate synthase; (**B**) Homotetrameric structure of Chorismate synthase (CS) from *Streptococcus pneumoniae* (PDB code 1QXO), where each monomer is shown in a different color; (**C**) Monomer of CS showing the flavin mononucleotide (FMN) and with 5-enolpyruvylshikimate 3-phosphate (EPSP); (**D**) Active site of CS with EPSP bound. The N5-position of the flavin is at the proper distance and orientation to act as the active site base. Residues that have been shown to be important in catalysis are shown. D339 is predicted to activate a water molecule (red sphere) for deprotonation of the N5-position of the FMN. H110 is close to the N1-O1 locus, where it can donate a proton to form the neutral reduced flavin as proposed. The other residues are involved in substrate binding or proton donation during phosphate release [26–28].

Chorismate synthase (CS) is one of the enzymes in the shikimate pathway, leading to the production of aromatic amino acids and other metabolites in prokaryotes, plants, and parasites such as *Plasmodium falciparum*[17]. Because this pathway is absent in mammals, enzymes in the shikimate pathway represent potential drug targets for the identification of novel antibiotics, antiparasitic agents, and herbicides [17,18]. CS catalyzes the conversion of 5-enolpyruvylshikimate 3-phosphate (EPSP) to chorismate (Figure 1). This reaction involves the *anti*-1,4-elimination of the 3-phosphate and cleavage of the C6-(pro*R*)-hydrogen from EPSP with no overall net redox change [19–21]. However, CS has an absolute requirement for the reduced form of the FMN cofactor [22]. The mechanism of action of CS has been the subject of extensive studies, as it is a potential drug target and for its requirement of reduced FMN in this non-redox reaction, which suggests a novel mode of action for the cofactor [17,23]. A concerted mechanism has been ruled out for the CS reaction, with both theoretical and experimental results showing that a concerted 1,4 elimination should yield a *syn* stereochemistry, which is inconsistent with the observed overall *anti*-stereochemistry of the product [20,21,24]. The order of bond cleavage in CS has been studied by transient kinetic analysis and it was determined that cleavage of the C3-O bond precedes rupture of the C6-(pro-*R*)-H bond [25].

Using a variety of flavin analogues, it was shown that EPSP binding triggers conformational changes in the enzyme that make the microenvironment of the reduced anionic flavin more hydrophobic, promoting protonation at the N1 position of the isoalloxazine ring to form a neutral reduced FMN. Under this condition the formation of a flavin semiquinone is favored [29]. Neutral reduced FMN was proposed to be the intermediate observed during rapid reaction kinetic analysis for this reaction. This rate of intermediate formation is faster than all the other chemical steps [25,30]. Observation of the neutral reduced FMN provided further experimental data of the role of the reduced FMN in catalysis, possibly in an electron transfer step [25]. The conformational change and the formation of a long-lived flavin semiquinone are also observed during rapid reaction kinetic experiments with the substrate analog (6*S*)-6-fluoro-EPSP [23,31]. The observed conformational change that leads to the protonation of the N1-position of the flavin was termed “substrate induced cofactor activation” by Macheroux and co-workers since the substrate is required to protonate the flavin [29].

**Scheme 2:**
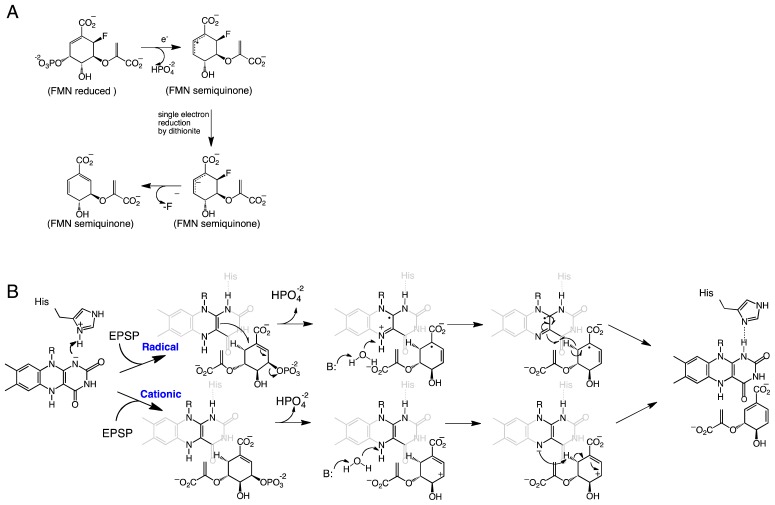
(**A**) Proposed mechanism of defluorination of (6*R*)-fluoro-EPSP by CS [31]. (**B**) Proposed radical and cationic mechanisms for CS reactions. In *Streptococcus pneumoniae*, the His residue corresponds to His110 [28]. The active site base that activates the water molecule has been shown to be an aspartate residue.

Kinetic studies with (6*R*)-fluoro-EPSP further supported the role of the flavin radical in catalysis. Dithionite-reduced CS was able to convert (6*R*)-fluoro-ESPS to chorismate [29]. Additionally, the reaction resulted in the formation of a stable product-bound flavin semiquinone that was essentially irreversible. Bornemann and co-workers proposed the mechanism shown in Scheme 2A for the reaction of CS with this analog [31]. In this mechanism, a single electron from the flavin is transferred to (6*R*)-6-fluoro-EPSP promoting the elimination of the phosphate group and forming an allylic radical [31]. The reaction stalls at this step since elimination of the fluoride atom at the C6-position is not favorable. Both the flavin radical and the allylic radical intermediate are stabilized by the protein, permitting reduction of the allylic radical by dithionite and forming the allylic anion, which is capable of fluoride elimination to form chorismate [31]. A flavin semiquinone intermediate was also observed during transient kinetic experiments with (6*S*)-6-fluoro-EPSP [23]. Participation of a flavin radical in the CS reaction was further supported by experiments with FMN analogs, which showed that activity was only observed with CS substituted with 1-deazaFMN and not with 5-deazaFMN [31].

The crystal structure of CS from the pathogenic bacterium *Streptococcus pneumoniae* was solved in the presence of EPSP and oxidized FMN at 2.0 Å resolution [28]. The structure revealed a novel β-α-β fold, consisting of four helices, the helical domain, sandwiched between two four-stranded antiparallel beta sheets [28]. The crystal structure of CS predicts that the enzyme forms a homotetramer, consistent with ultracentrifugation and size exclusion chromatography studies [32]. The active site is composed of structural components derived from residues from the second β-sheet and the helical domain. Explanation for several important features were revealed by the crystal structure, including the dramatic reduction in the *K*_d_ value for the flavin cofactor in the presence of ESPS (from 30 μM to 20 nM) [33]. Oxidized FMN interacts with CS, mainly with its ribotyl moiety and ~90% of the isoalloxazine ring. EPSP binds to the *re*-face of the flavin and covers the rest of the exposed flavin, promoting additional conformational changes that cause further interactions between FMN and CS [28]. The net result is an almost complete exclusion of the FMN from the solvent upon ESPS binding, which would reduce the off-rate of the cofactor, causing the observed decrease in the *K*_d_ value [33]. It was also observed that His110 is located in close proximity to the N1 atom of the flavin where it could donate a proton to form the neutral reduced FMN upon EPSP binding. Site-directed mutagenesis of this conserved residue to an alanine in the *Neurospora crassa* enzyme results in a 20-fold decrease in activity, supporting its role in catalysis [26].

EPSP binds at the *re*-face of the flavin and interacts with several arginines and histidine residues. The binding conformation of EPSP provides a very important piece of information regarding the chemical mechanism of CS. It clearly shows that there is no amino acid residue in close proximity to the C6-position on EPSP that can act as a base. Surprisingly, the only candidate is the N5 atom of the flavin, which is at 3.5 Å and in close enough proximity to act as the base (Figure 1D) [28]. This observation prompted MacLean and Ali to propose two mechanisms involving the N5-position in C6-(*pro*-*R*) hydrogen bond cleavage, either as a hydrogen or a proton, depending on whether a radical or a cationic intermediate initiates the reaction, respectively (Scheme 2B) [28]. The proposed role of the flavin as an active-site base requires the deprotonation of the N5 of FMN to facilitate C–H bond cleavage in EPSP. This deprotonation step was proposed to be mediated by a conserved aspartate, which is located below the flavin and can activate a water molecule to deprotonate the N5-position [27,28]. Site-directed mutagenesis of this conserved aspartate in *Neurospora crassa* (Asp367) to either alanine or asparagine had no effect on binding of ESPS or the oxidized FMN. However, the activity decreased 300–600 fold, providing strong support for the role of this invariant aspartate residue as the active-site base that primes the N5 of the reduced FMN for C–H bond cleavage [27].

The structural and mechanistic data clearly demonstrate that the chemical mechanism of CS involves (1) binding of the anionic reduced FMN, (2) protonation of the N1-positon of the flavin by a histidine residue (His110 in *S. pneumoniae*) upon substrate binding, the so-called “substrate induced cofactor activation”, and (3) nonconcerted bond cleavage with the C3–O bond cleaving first. Although the steps leading to bond cleavage are still a matter of discussion, the experimental data favors the radical intermediate (Scheme 2B). For example, studies with C6-fluoro-EPSP clearly demonstrate the presence of a flavin radical in catalysis. Furthermore, the semiquinone preferentially deprotonates at the N5 position with a pK_a_ value of ~8 [34]. The resulting flavin radical can abstract a hydrogen from the C6-*pro*-*R*-H position to form chorismate as shown in Scheme 2B. Other variations of the radical mechanism in which the flavin abstracts a proton from the C6-pro-*R* hydrogen have been proposed by Macheroux and co-workers [35].

## 3. Type II Isopentenyl Diphosphate/Dimethylallyl Diphosphate Isomerase

**Figure 2:**
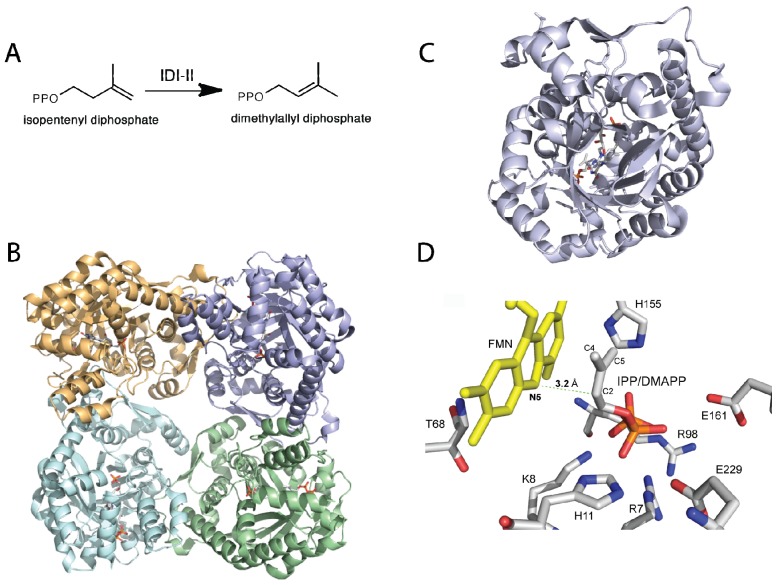
(**A**) Reaction catalyzed by isopentenyl diphosphate/dimethylallyl diphosphate isomerase (IDI)-II; (**B**) Cartoon representation of IDI-II from *Sulfolobus shibatae* (PDB code, 2ZRY), showing each monomer in a different color; (**C**) Top view of a monomer of IDI-II showing the FMN bound at the center of the TIM barrel; (**D**) Residues in the active site predicted to play a role in substrate binding. The position of IPP in the active site shows that only the flavin N5 is in proper orientation to function as an acid/base during catalysis.

Isopentenyl diphosphate/dimethylallyl diphosphate isomerase (IDI) catalyzes the isomerization of isopentenyl pyrophosphate (IPP) to dimethylallyl pyrophosphate (DMAPP) (Figure 2). This reaction is essential for bacterial growth, including in many human pathogens. There are two unrelated families of IDI enzymes, which are grouped as either type I or type II [36]. Type I IDI (IDI-I) enzyme are divalent metal ion dependent requiring Zn^2+^ and Mg^2+^ for activity [37,38]. The chemical mechanism of IDI-I has been studied in detail and involves acid/base chemistry [37,39,40]. The reaction is believed to involve the formation of a carbocation intermediate originating from the protonation of the double bond in IPP by a glutamate residue. The isomerization reaction is completed by action of a conserved cysteine residue that abstracts a proton at the C2 position [40]. Type II IDI (IDI-II) enzymes are FMN dependent, that despite catalyzing a non-redox reaction, require NAD(P)H to reduce the FMN for effective catalysis [9,16]. IDI-II enzymes are found in archea and bacteria, including several human pathogens. It has been determined that the FMN is reduced by the stereospecific transfer of the *pro*-S hydrogen of NAD(P)H and the reduced form of IDI-II can undergo multiple catalytic cycles [41]. Additionally, it was shown that the flavin semiquinone was stabilized in the IDI-II-IPP complex and that substitution of the FMN by 5-deazaFMN yielded an inactive enzyme. In contrast, IDI-II substituted with 1-deazaFMN retained wild-type activity [41]. These results were consistent with a single electron transfer step during catalysis. However, studies with cyclopropyl-IPP and epoxy-IPP analogues did not support the presence of an FMN radical in the catalytic cycle of IDI-II [42]. IDI-II was able to catalyze the isomerization of cyclopropyl-IPP to cyclopropyl-DMAPP, while epoxy-IPP was a potent irreversible inhibitor (*K*_i_ 1.4 μM). The mechanism of inactivation involves the nucleophilic addition of epoxy-IPP to the FMNH_2_[42,43]. There was no evidence of radical rearrangement with either substrate analogue. The mechanism of inactivation was determined by solvent isotope effect studies to be initiated by the protonation of the epoxide group in epoxy-IPP by the lack of deuterium in the flavin-inhibitor adduct, since proton addition at the double bond of epoxy-IPP would have retained a deuterium atom [42]. Diene- and fluorinated-IPP analogues were also found to be irreversible inhibitors of IDI-II, and spectroscopy and mass spectrometry analyses of the flavin-inhibitor adduct were consistent with the site of covalent addition being at the N5 position of the flavin [43]. These results are consistent with a mechanism involving a protonation-deprotonation mechanism for IDI-II, as had been established for IDI-I [37,39,40,43]. Further evidence was obtained from deuterium isotope effect studies where a *^D^V* value of 1.8 was observed using (*R*)-[2-^2^H]-IPP, indicating that C2-H bond cleavage is partially rate limiting [44]. Stopped-flow kinetic analysis failed to identify the presence of a flavin-IPP radical intermediate in this reaction, even with deuterated substrate. Electron paramagnetic resonance analysis of photoreduced IDI-II in complex with IPP provided evidence of significant quantities of FMN radical; however, no signal of a substrate radical pair was detected. This indicated that the observed flavin radical might not be catalytically relevant [44].

UV-visible spectrophotometric studies demonstrated that the neutral reduced FMN accumulated upon substrate binding, and the accumulation and decay was kinetically competent. When (*R*)-[2-^2^H]-IPP was used as the substrate, an isotope effect value of 2.3 was measured on the decay of this intermediate [44]. Identification of the covalent adduct between the inhibitors and FMN, together with the lack of a radical pair in the enzyme FMN semiquinone/IPP complex, and the observed primary and solvent isotope effects, provide cumulative evidence against a radical intermediate and favor an acid/base chemical mechanism for IDI-II as determined for the IDI-I enzymes. Liu and co-workers have proposed several mechanisms, all involving the neutral reduced FMN playing various roles including charge stabilization, acting as either an acid or a base, or functioning as both an acid and a base [44]. A role for the reduced FMN in charge stabilization of the 3° carbocation intermediate is not supported by the experimental data that show that the neutral reduced flavin accumulates during turnover [44]. In contrast, the possibility of an acid-base functionality of the flavin is supported by several experimental results. As mentioned, the neutral reduced FMN is catalytically competent and in this form the p*K*_a_ of the N5 atom is lowered by protonation of the N1 position of the FMN, and possibly by hydrogen bond donation by an active site residue. The flavin could act as a base and form a zwitterionic 5,5-dihydro-N5-FMN upon abstraction of the *pro*-*R*-C2- proton [44,45].

Although the mechanistic data strongly support an acid-base chemical mechanism in IDI-II enzymes, more data was required in support of these novel roles for flavins in catalysis. In particular, information was needed on the structure of the active form of the enzymes with reduced flavin and in complex with substrate or product. In a recent publication, Hemmi and co-workers were able to solve the structure of IDI-II from the archeon *Sulfolobus shibatae* in both the oxidized and reduced forms, as well as in complex with substrate [46]. The enzyme is a homotetramer that contains a triose-phosphate isomerase (TIM) barrel fold consistent with other IDI-II structures [47]. Structural differences between the oxidized and reduced forms were only observed at the active site, where a threonine residue (Thr68) is in hydrogen-bonding distance of the N5 atom of the flavin in the oxidized form. In the reduced form, it is twisted away from the flavin and the backbone carbonyl oxygen of Met67 is in close proximity to the N5 atom of the flavin. Substrate binding also promotes conformational changes in the active site, precluding solvent access in the bound form [46]. Upon substrate binding, an α-helix (α-4, residues 95–102) and a loop region between helices 8 and 9 (residues 162–177) change orientation into the closed conformation. These changes are independent of the redox state of the flavin, as they are observed in both the oxidized and reduced structures. As was previously proposed and expected, based on the experimental data, the substrate binds close to the isoalloxazine ring of the flavin (~4 Å) [46,47]. In the active site, several residues including Arg7, Lys8, Ser9, Arg98, His155, and Gln161, are in close proximity to interact with the substrate. Mutations of these and 8 other charged or polar conserved residues to alanine produced mutant enzymes, which contained bound flavin, and were able to oxidize NAD(P)H and bound substrate with similar affinities to the wild-type enzyme. However, the catalytic efficiency of most of the mutant enzymes was much lower (~1% to ~35%) compared to the wild-type enzyme, indicating that these residues play a role in catalysis [46].

**Scheme 3:**
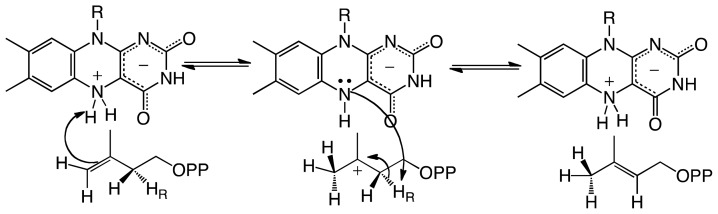
Chemical mechanism of IDI-II where the N5 of the FMN is proposed to act as an acid and base in the formation of dimethylallyl pyrophosphate.

Although results from alanine scanning indicated that the conserved residues in the active site of IDI-II play some role in catalysis, perhaps in transition state stabilization. However, the resulting mutant enzymes retain significant activity, indicating that none function as the active site acid-base. Based on the position of the IPP/DMAPP observed in the structure, the only other possible candidate that can act as the acid-base is the flavin cofactor; specifically, the N5 atom of the FMN, which is ~3.6 Å from the C4 carbon and ~3.2 Å from the C2 carbon of IPP/DMAPP (Figure 2). The binding mode of the substrate provides further evidence that the reduced FMN plays a role as an acid-base in the IDI-II reaction. Unno *et al.* proposed that the structure of the IDI-II bound to its substrate supports a mechanism in which the N5 loci of the zwitterionic FMN might act as an acid forming the 3° carbocation, which is stabilized by the negative charge of the resulting deprotonated reduced flavin. The N5 atom is also in a proper orientation to act as a base to abstract the proton at the C2 position. The proposal of the zwitterionic FMN playing a role as an acid/base in IDI-II emerges as an attractive possibility since the p*K*_a_ of the N5 in this form of the flavin is lowered to a value of ~4 [45]. The proposed role of the N5 loci acting as an acid is consistent with the mechanism of inhibition by epoxy-IPP where opening of the oxirane ring is initiated by protonation of the epoxide oxygen, which becomes a target for a nucleophile—in this case, the N5 nitrogen of the FMN [42,43]. More recently, Liu and coworkers utilized kinetic linear free energy analysis with flavin analogs to demonstrate that the flavin–N5 likely functions as an acid/base in the reaction [48]. The same group has shown that when the reaction is performed in deuterium oxide and tritiated substrates are used, the stereochemistry of the product supports a direct role of the reduced flavin in the protonation of IPP [49]. The accumulated mechanistic and structural data provide strong support for the reduced flavin cofactor acting both as an acid and base to catalyze the suprafacial protonation and deprotonation in the IDI-II enzymes. Together, the data strongly support the mechanism shown in Scheme 3.

## 4. UDP-Galactopyranose Mutase

**Figure 3:**
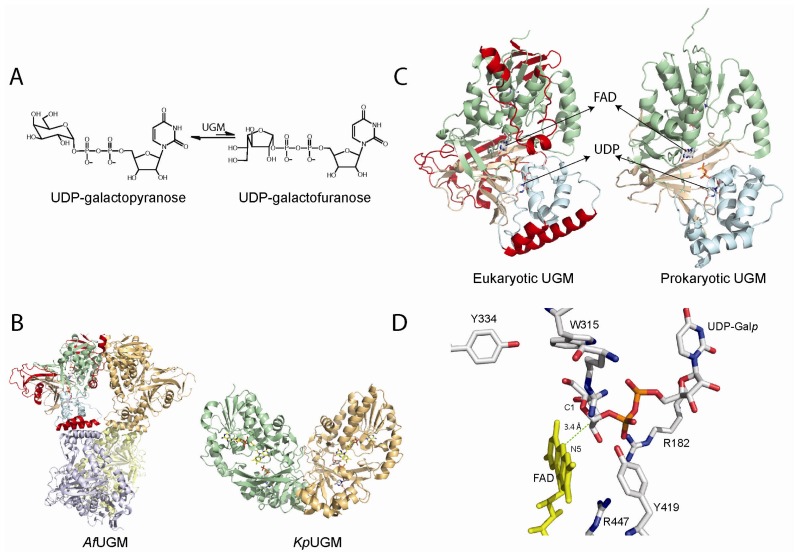
(**A**) Reaction catalyzed by UDP-galactopyranose mutases (UGMs); (**B**) Cartoon representation of the tetrameric fungal (PDB code 3UTH) and dimeric bacterial (PDB code 3GF4) UGMs; (**C**) Monomers of UGMs showing the flavin adenine dinucleotide (FAD) binding domain (green), helical domain (blue), and beta sheet domain (gold). The additional secondary structures in the eukaryotic UGMs are highlighted in red; (**D**) Active site of eukaryotic UGMs showing residues predicted to interact with UDP-Galp. The interaction between the sugar C1 atom and the N5 of the FAD is depicted with a green dotted line.

Biosynthesis of galactofuranose (Galf) involves the conversion of UDP-galactopyranose to UDP-galactofuranose (UDP-Galf) by the enzyme UDP-galactopyranose mutase (UGM) (Figure 3). Galf is then transferred to the corresponding glycoconjugates by UDP-Galf transferases [50,51]. There are numerous UDP-Galf transferases (e.g., ~20 in *Trypanosoma cruzi*) that are required for the transfer of Galf to the final sugar acceptors at the cell wall and cell surface of bacteria, fungi and parasites. Thus, UGM plays a central role in the biosynthesis of Galf in microbes as it is the only source of this unusual sugar [50]. The importance of this enzyme has been demonstrated in *Mycobacterium smegmatis*, where deletion of the UGM gene has shown that it is essential for growth [52]. Similarly, deletion of the UGM gene in the fungi *Aspergillus fumigatus* results in attenuated virulence and increased sensitivity to antifungal agents due to a decrease in cell wall thickness in the mutant strain [53]. The importance of this enzyme in kinetoplastids has also been validated by demonstration that deletion of the UGM gene leads to greatly reduced virulence in *Leishmania major*[54]. These data clearly indicate that UGM is a good drug target as it plays a role in pathogenesis of bacteria, fungi, and parasites, which cause diseases such as tuberculosis, aspergillosis, leishmaniasis, and Chagas’ disease [52,55–57]. Furthermore, homologs of UGM are absent in humans, suggesting that drugs specific for this enzyme should have low host-specific toxicity.

The structures of the bacterial and eukaryotic UGMs have been solved in both the inactive oxidized and active reduced states [58–62]. The overall fold is very similar between these two classes of enzymes and belongs to the α/β structural class and is composed of two domains, a classical FAD Rossmann domain and a novel 5-helical domain. A cleft is formed between these two domains and the isoallozaxine ring of the FAD is located at the end of the cavity [58–61]. In the prokaryotic enzymes there are several conserved residues in this region and site-directed mutagenesis has shown that these residues are important in catalysis [61,63]. These active site residues are not totally conserved in the eukaryotic enzyme, which is not surprising since these enzymes share relatively low sequence identity (<18%). Eukaryotic UGMs are ~50–100 amino acids longer than the prokaryotic enzymes [50,55]. This feature renders these enzymes with additional secondary structure, which plays a role in quaternary structure and catalysis. In general, prokaryotic UGMs are thought to function as homodimers [61]. In contrast, *A. fumigatus* UGM (AfUGM) has been shown to function as a homotetramer while *L. major* and *T. cruzi* UGMs are monomeric enzymes (Figure 3) [64,65]. Another consequence of the additional amino acid sequence found in the eukaryotic UGMs is the presence of two highly dynamic loop regions called the 180 flap (179–187 in AfUGM) and the 200 flap (203–209 in AfUGM), which move 11–13 Å [58]. These mobile flaps have been observed in the structures of both AfUGM and TcUGMs. In the prokaryotic enzymes, a loop (167–177 in *Klebsiella pneumoniae*) has also been shown to have similar flexibility as the 180 flap in eukaryotic UGMs [60].

The mechanism by which UGM catalyzes the conversion of UDP-Galp to UDP-Galf is unprecedented. The overall reaction does not involve the reduction or oxidation of the galactose substrate, yet, the reduced form of the FAD cofactor is required [57,61,65–67]. Using oxygen positional isotope exchange (PIX), it was demonstrated that the glycosidic bond is broken during catalysis [68,69]. Potentiometry studies suggest that the semiquinone form of the flavin is formed and stabilized by substrate binding [70]. It was also shown that 5-deaza-flavin substituted prokaryotic UGM is inactive, suggesting that an electron transfer step is necessary for catalysis [71]. Kiessling and coworkers demonstrated that a covalent substrate-FAD adduct is formed between the anomeric carbon and the N5 atom of the flavin in *K. pneumoniae*[72]. The presence of such an intermediate has also been shown in eukaryotic UGMs [64]. These data led to two proposals describing the mechanism by which UGM catalyzes the FAD-dependent ring contraction to form UDP-Galf. One mechanism invokes the flavin as a nucleophile that attacks the anomeric carbon to displace UDP. The other mechanism involves a single electron transfer from the reduced flavin to a postulated oxocarbenium sugar intermediate followed by the formation of a flavin-sugar adduct [9,55,61,70]. In both mechanisms, the covalent flavin-sugar adduct is important for opening and recyclization of the galactose ring. Previous mechanistic proposals invoked a 1,4-anhydrogalactopyranose as an intermediate in the reaction, but this hypothesis was ruled out by lack of activity of the UGM with chemically synthesized 1,4-anhydrogalactopyranose and UDP [73]. Experiments with 2-F or 3-F UDP-galactose showed that the chemical mechanism does not involved oxidation at these positions [69,74].

The structure of UGM in complex with UDP-Galp provided strong evidence for a direct role of the flavin cofactor in catalysis as a nucleophile (Figure 3). In this structure, the anomeric carbon of Galp is located adjacent to the N5 of the flavin at ~3.4 Å [58,62,75,76]. Furthermore, under rapid reaction kinetic conditions, reaction of reduced *T. cruzi* UGM with UDP-Galf occurred in the absence of a detectable flavin semiquinone, only the formation of a species characteristic of a flavin iminium ion was observed [64]. These results do not support the mechanism involving electron transfer steps. Together, the identification of the flavin adduct, rapid reaction kinetic experiments, and the structure of the UGM/UDP-Galp complex strongly support an S_N_2 displacement of the UDP by a direct attack of the N5 of the flavin to the anomeric carbon of UDP-Galp. However, the possibility of the adduct forming by an attack of the flavin to an oxocarbenium ion intermediate (S_N_1) could not be ruled out. Recently, flavin analogues were used to determine kinetic linear free energy relationship of *k*_cat_ to the nucleophilicity of the N5 of the flavin. The results show a slope, ρ of -2.4, which is consistent with the direct attack of the N5 of the flavin to the UDP-Galp [77].

**Figure 4:**
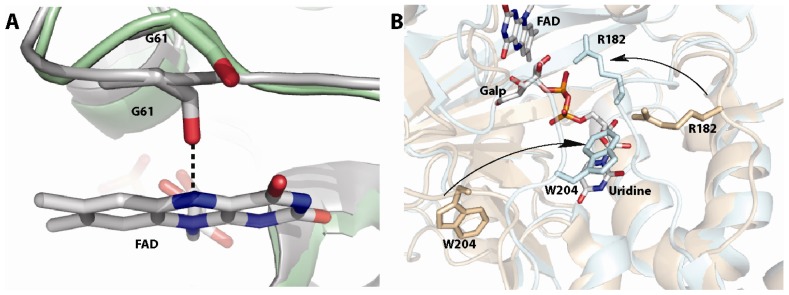
(**A**) Redox mediated motions of the histidine loop (green depicts the oxidized form and gray the reduced form). The figure shows the motion of the backbone carbonyl of G61 toward the N5 of the flavin upon reduction. This interaction is important for activity [59]; (**B**) Closing of the 180 and 200 flaps upon substrate dining in Af UGM (free protein shown in gold and substrate bound shown in cyan) [58]. These loops move 11–13 Å.

Another unknown regarding the mechanism of UGMs was the source of reducing equivalents. A common redox partner for flavoenzymes is NAD(P)H, however, the amino acid sequence and 3D-structure did not show or predict a canonical NAD(P)H binding domain. Thus, how are these enzymes activated? Initial experiments showed that bacterial enzymes were reduced by NAD(P)H, although the reaction required excess reduced coenzyme (20 mM) and very long incubation periods (>10 min) [78]. In contrast, studies with eukaryotic UGM show that these enzymes are effectively reduced by NADPH with a rate constant for flavin reduction (*k*_red_) of 0.6 s^−1^ and a *K*_d_ value of 98 μM [64]. Furthermore, it was shown that eukaryotic UGMs can stabilize the reduced form of the flavin, under aerobic conditions, such that for every NADPH utilized it can turnover ~1000 times [64]. Thus, at least for eukaryotic UGMs, NADPH appears to be the physiological partner. The mechanism of stabilization is shown to include conformational changes in a loop region known as the histidine loop, which upon reduction brings the residues Gly60, Gly61, and His62 into hydrogen bonding distance to the flavin cofactor, including hydrogen bonding between the backbone carbonyl of Gly61 and the FAD-N5 (Figure 4A). Mutation of residues in this loop decrease the activity of the protein by 10–100 fold [59].

**Scheme 4:**
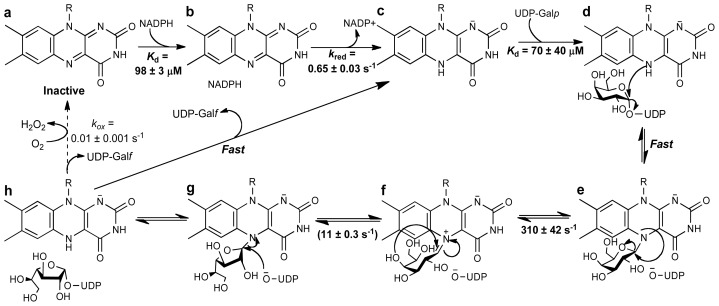
Chemical mechanism for eukaryotic UGMs. Activation of the flavin to the reduced form is achieved by reaction with NADPH (**a**–**c**). Binding of UDP-Galp to the reduced enzyme places the anomeric carbon in close proximity to the N5 of the flavin, such that a flavin-sugar adduct is formed after a direct N5-attack and UDP-displacement (**d**,**e**). Formation of an iminum ion allows ring opening and formation of galactofuranose after recyclization (**f**,**g**). Attack of UDP to the C1 releases the Galf from the FAD and forms UDP-Galf. Product release has been shown not to be rate limiting. The reaction with molecular oxygen is very slow, allowing for ~1000 rounds of UDP-Galf formation before the enzyme is oxidized, and another NADPH molecule is needed. The binding affinities and kinetic rate constants are those determined for *Trypanosoma cruzi* UGM [64].

Steady-state and stopped-flow kinetic analyses, in combination with the structural and mechanistic data determined in eukaryotic UGMs, have allowed the complete characterization of the kinetic mechanism of these enzymes (Scheme 4). In this reaction the flavin functions as a nucleophile leading to the formation of a flavin-sugar adduct, which functions as a scaffold for the ring opening/contraction steps. The unique fold of UGMs modulate this activity. In addition, significant conformational changes important for keeping solvent from entering the active site, for keeping UDP bound, and for maintaining the active reduced form of FAD are observed in this enzymes. This information will aid in the development of inhibitors with a potential impact in the fight against bacterial, fungal, and parasitic human pathogens.

## 5. Alkyl-dihydroxyacetonephosphate Synthase

**Figure 5:**
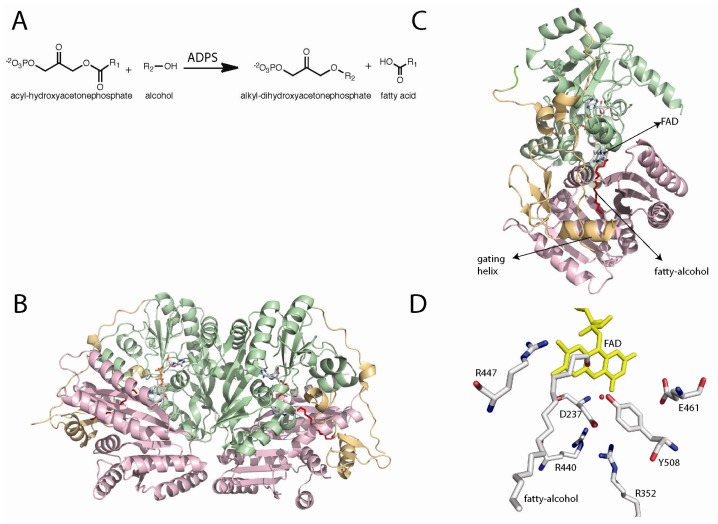
(**A**) Reaction catalyzed by alkyl-dihydroxyacetonephosphate synthase (ADPS); (**B**) Dimer of ADPS; (**C**) Location of the FAD and fatty alcohol binding sites in the monomer structure; (**D**) Putative active site of ADPS. Site directed mutagenesis has shown that R352, Y508, and R447 are important for catalysis [86].

Alkyl-dihydroxyacetonephosphate synthase (ADPS) catalyzes the formation of an ether bond in the biosynthesis of ether phospholipids. This reaction involves the exchange of an acyl chain at the C-1 position of acyl-dihydroxyacetonephosphate, with a fatty alcohol forming an ether linkage in alkyl-DHAP (Figure 5) [79–81]. This reaction does not involve net redox change and like the other enzymes discussed in this review, ADPS requires a flavin cofactor for catalysis; however, ADPS requires the oxidized form of the flavin and not the reduced form [82]. The products of the ADPS reactions, ether phospholipids, are major components of cell membranes and are involved in vesicle trafficking and signal transduction [80,81,83,84]. Severe human diseases have been shown to be the direct result of mutations of the ADPS gene [80,85]. Rhizomelic chondrodysplasia punctata (RCDP) type 3 is caused by an inborn deficiency of ADPS [80]. Persons with RCDP suffer from severe growth and developmental problems and, ultimately, die before reaching puberty [85].

The amino acid sequence of ADPS contains a signal sequence for its localization to peroxisomes [87–90]. Early mechanistic work was done with microsomal fractions and solubilized enzyme [91,92]. It was shown by tritium exchange experiments that the *pro*-*R*-H at the C1 position was abstracted and exchanged with the solvent [93–95]. Additionally, it was demonstrated by isotope labeling experiments that both of the oxygens on the fatty acid are removed during the acyl cleavage and that the oxygen in the ether bond is donated by the alcohol substrate [96,97]. A more detailed characterization was possible due to the expression and purification of large quantities of recombinant ADPS from guinea pig in *Escherichia coli*[87]. Steady-state kinetics analysis indicated that ADPS follows a ping-pong mechanism, where the fatty acid is released before the second substrate, the fatty alcohol, binds and reacts to form the final alkyl-DHAP product [87,92]. It was suggested, based on amino acid sequence conservation, that ADPS might be a member of the flavin dependent vanillyl-alcohol oxidase family [98]. Van den Bosch and co-workers demonstrated that the recombinant form of ADPS contains a tightly bound FAD [82]. Stopped-flow experiments were used to show that mixing acyl-DHAP with oxidized enzyme leads to flavin reduction. This reduced species was oxygen stable and the rate of its formation was catalytically competent. The enzyme could be oxidized by addition of the second substrate, the alcohol. These results clearly demonstrate that the flavin cofactor plays a role in catalysis [82].

The X-ray crystal structure of *Dictyostelium discoideum* ADPS was solved at 2.1 Å resolution [86,99]. The structure shows a similar FAD-binding domain to the members of the vanillyl-alcohol oxidase family of flavoenzymes, as predicted by the amino acid sequence analysis [98]. The structure was solved in the presence of an aliphatic molecule with similar dimensions to those of palmitoyl alcohol. This molecule was not added during protein production, purification, or crystallization. The adventitious binding of the fatty alcohol clearly shows a substrate binding channel that extends from the surface of the protein toward the active site [86]. This channel contains the proper dimensions to fit an aliphatic chain of 16 carbons, which provides a structural explanation for the observed substrate preference for palmitoyl-DHAP and hexadecanol [81]. Entrance to the active site is controlled by an additional domain found only in ADPS. This domain is composed of a β-sheet and an α-helix. Mattevi and co-workers proposed that this domain controls substrate access to the active site. The helix closes the channel upon substrate binding, the “in” conformation where the helix is well defined, and in the substrate free enzyme the helix is in the “out” position, where the helix is observed in various conformations [86]. This helix is called the “gate keeping helix”.

**Scheme 5:**
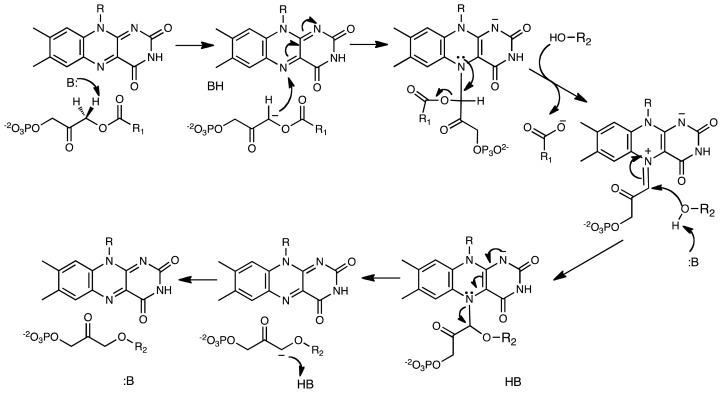
Reaction catalyzed by alkyl-dihydroxyacetonephosphate synthase (ADPS). In this mechanism, an active site base abstracts a proton forming a carbanion intermediate that reacts with the N5 of the flavin forming an adduct. With formation of a flavin-DHAP-iminum ion, the fatty acid is eliminated and the fatty alcohol is added to the activated carbon. In this reaction, the oxidized flavin functions as an electrophile and a scaffold.

The mechanism shown in Scheme 5 was proposed by Mattevi and co-workers as a working model for the ADPS catalyzed reaction. In this mechanism, acyl-DHAP accesses the active site through the channel, positioning the DHAP moiety in close proximity to the flavin. In the structure, Tyr508 is located within hydrogen-bonding distance to the hydroxyl group of the substrate. This residue is the best candidate to catalyze the abstraction of the pro-*R* proton at the C1 position of DHAP. The role of Tyr508 as the active-site base is supported by mutagenesis studies that show that this residue is essential for catalysis [86]. Proton abstraction results in the formation of a carbanion that can be stabilized by delocalization of the charge onto the carbonyl group. This carbanion is capable of attacking the electrophilic N5 of the oxidized flavin, forming a covalent adduct (Scheme 5).

Cleavage of the fatty acid group occurs with formation of a flavin-DHAP-iminium intermediate. The role of the flavin as an electrophile is consistent with the kinetic ping-pong mechanism, which requires the release of the fatty acid prior to the reaction with the fatty-alcohol. This is also consistent with isotope labeling data that demonstrated that both oxygens in the fatty acid are retained, and stopped-flow data that shows the reduction of the flavin upon addition of acyl-DHAP [82,96,97]. The fatty-alcohol can then bind and react with the activated C1-carbon in the flavin iminium intermediate (Scheme 5). Cleavage of the flavin-product adduct, leads to the alkyl-DHAP and the free oxidized flavin. During the catalytic cycle, binding and release of substrates and products is mediated by the gating helix [86]. Experimental evidence of the function of oxidized flavins as nucleophiles in flavoproteins has been demonstrated in nitroalkane oxidase (NAO) from *Fusarium oxysporum*. NAO, as isolated from *F. oxysporum,* is not active and the flavin spectrum shows characteristics of an N5-flavin adduct [100,101]. Mass spectrum analysis demonstrated that this adduct was the product of the covalent addition of nitrobutane to the FAD cofactor [101]. Furthermore, the covalent substrate-flavin intermediate in NAO has also been shown by X-ray crystallography [102,103]. The functionality of the flavin cofactor as an electrophile in reactions that stabilize a carbanion intermediate appeared to be well supported by experimental data and, therefore, it is very likely that such a mechanism is also utilized by ADPSs [13,86,104].

## 6. Concluding Remarks

Flavoenzymes that catalyze reactions with no next redox change have represented a challenging but rewarding area of investigation for flavoenzymologists. These enzymes have been shown to tune the flavin cofactor to add “new” functionalities. In the reaction catalyzed by CS, the mechanism is still very much debated. However, it is postulated that the flavin transfers one electron to promote C–O bond cleavage and C–H bond cleavage by a radical mechanism. In this reaction, the flavin is proposed to act as a base/hydrogen atom abstractor for C–H bond cleavage after breaking of the C–O bond. In UGMs, the role of the flavin as a nucleophile attacking the anomeric carbon of galactose has been well established. This mixed role for the flavin as an electron donor and nucleophile or as a base, is modulated in CS and UGM by novel protein folds. IDI-II has evolved by convergent evolution to catalyze the same reaction as IDI-I, but without the aid of metal ions or protein-derived functional groups. Instead, IDI-II employs novel dual flavin functionalities, acting both as an acid and as a base. In reactions where an intermediate must be held in place to permit the interaction with a second substrate, such as in ADPS, the oxidized flavin acts as an electrophile forming a flavin-DHAP-iminum ion. This intermediate is required for the addition of the fatty-alcohol to form alkyl-DHAP. This functionality of the flavin cofactor as a molecular scaffold to hold the intermediate in catalysis is also observed in UGM. In UGM, the covalent intermediate promotes the opening of the galactose ring and activation of the C1 carbon to allow ring contraction. In summary, structural and mechanistic information has accumulated to provide strong evidence of various noncanonical roles of flavin cofactors in nature: acid/base and electrophile/nucleophile chemistries and covalent intermediates. Our understanding of the chemical mechanism, including the structure of these intermediates, will have a significant impact on human health and agriculture, as all of the enzymes discussed herein are important drug targets against bacteria, fungi, and parasites, are potential herbicide targets, or are involved in the development of human diseases.

## Figures and Tables

**Table 1:** Novel roles of flavoenzymes involved in no net redox change reactions.

Role of flavin cofactor	Flavin redox state *	Enzymes
Acid/base chemistry	FMNred	Type II isopentenyl diphosphate isomerase
Radical/base	FMNred	Chorismate synthase
Nucleophile/scaffold	FADred	UDP-galactopyranose mutase
Electrophile/scaffold	FADox	Alkyl-dihydryoxyacetonephosphate synthase

* red = reduced; ox = oxidized.
